# lncRNA XIST regulates proliferation and migration of hepatocellular carcinoma cells by acting as miR-497-5p molecular sponge and targeting PDCD4

**DOI:** 10.1186/s12935-019-0909-8

**Published:** 2019-07-29

**Authors:** Yixi Zhang, Zebin Zhu, Shanzhou Huang, Qiang Zhao, Changjun Huang, Yunhua Tang, Chengjun Sun, Zhiheng Zhang, Linhe Wang, Huadi Chen, Maogen Chen, Weiqiang Ju, Xiaoshun He

**Affiliations:** 1 Organ Transplant Center, The First Affiliated Hospital, Sun Yat-sen University, No. 58 Zhongshan Er Road, Guangzhou, 510080 China; 2 Guangdong Provincial Key Laboratory of Organ Donation and Transplant Immunology, Guangzhou, China; 3 Guangdong Provincial International Cooperation Base of Science and Technology, Guangzhou, China; 4 Organ Transplant Center, The First Affiliated Hospital of USTC, Division of Life Sciences and Medicine, University of Science and Technology of China, Hefei, 230001 Anhui China; 5 Department of General Surgery, Guangdong General Hospital, Guangdong Academy of Medical Sciences, Guangzhou, China

**Keywords:** lncRNA XIST, PDCD4, miR-497-5p, Proliferation, Migration, Hepatocellular carcinoma

## Abstract

**Background:**

MicroRNAs (miRNAs) play a pivotal role in hepatocellular carcinoma (HCC) progression and have been confirmed to participate in the carcinogenesis and development of HCC. However, the relationship between miR-497-5p and HCC remains unclear.

**Methods:**

Kaplan–Meier curve analysis and the log-rank test were used to investigate the efficacy of miR-497-5p on overall survival (OS) and disease-free survival (DFS) in patients with HCC. According to in vitro experiments, programmed cell death 4 (PDCD4) was a target of miR-497-5p by the dual-luciferase activity assay. The efficacy of PDCD4 on cell proliferation and metastasis in HCC was examined by transwell assays, CCK-8 assays and reverse transcription quantitative PCR (RT-qPCR). Additionally, we conducted a luciferase activity reporter assay to confirm the interaction between lncRNA XIST and miR-49-5p. Then, to evaluate the relationship between lncRNA XIST and miR-497-5p, several mechanistic experiments, including qRT-PCR, Western blotting, transwell assays and tumor xenograft assays, were performed.

**Results:**

miR-497-5p was upregulated in HCC tissues, and high expression of miR-497-5p resulted in increases in tumor size and tumor number and a higher tumor-node-metastasis (TNM) stage and Edmondson grade in patients with HCC. Silencing miR-497-5p inhibited the proliferation and migration of HCC cells. PDCD4, which was downregulated in HCC tissues, was shown to be a target of miR-497-5p and was negatively correlated with the expression of miR-497-5p. lncRNA XIST was found to act as a miR-497-5p sponge and to regulate the level of PDCD4, which is targeted by miR-497-5p. lncRNA XIST was observed to be downregulated in the HCC tissues and positively correlated with the expression of PDCD4.

**Conclusions:**

Our findings reveal that the XIST/miR-497-5p/PDCD4 axis participates in HCC development and that XIST could be used as a biomarker of HCC.

## Background

Hepatocellular carcinoma (HCC) is one of the most deadly tumors in the world, especially in China [1] due to the high hepatitis B virus (HBV) infection rate [2–4]. Surgery and some other interventional therapies have greatly improved in recent years, but the outcomes of HCC patients remain poor [5]. Because of frequent recurrence and metastasis, HCC patients usually have poor prognosis [6]. Thus, exploring the mechanisms of HCC development is important for optimizing early diagnosis and treatment [7, 8].

Recent research has shown that the aberrant expression of noncoding RNAs (ncRNAs) is ubiquitous in different types of cancers, suggesting that ncRNAs play a key role in human carcinogenesis [9]. NcRNAs of less than 200 nucleotides are considered as small ncRNAs, i.e., microRNAs, while ncRNAs of more than 200 nucleotides are considered as long ncRNAs, i.e., lncRNAs [10]. Growing evidence suggests that the abnormal expression of lncRNAs is implicated in a variety of diseases, including cancer [11–13], and that some tumor-associated lncRNAs play key roles in the development and metastasis of HCC [14–16]. For example, lncRNA HULC [17], lncRNA EGFR [18], lncRNA HOST2 [19] and lncRNA Tim3 [20] accelerate HCC tumorigenesis and metastasis. However, lncRNA FTX [21] has been reported to inhibit HCC development and proliferation. lncRNA beta-Catm [16] is essential for the self-renewal of hepatocellular carcinoma stem cells and the proliferation of HCC tumors. MicroRNAs (miRNAs) constitute a group of small RNAs containing 18–25 nt. There is growing evidence that miRNAs are involved in various types of biological processes such as self-renewal, survival and tumor progression [22, 23]. In various studies, several miRNAs, such as miR-451, miR-128, miR-34 and miR-203, have been suggested to regulate cancer stemness and drug resistance in different types of cancer [24]. By targeting the 3′-untranslated regions (UTRs) of mRNAs, the expression of the target gene can be regulated posttranscriptionally, thereby affecting the regulation of cell proliferation, differentiation and apoptosis [1].

In the current study, we hypothesized that lncRNA X inactive-specific transcript (XIST) targets specific miRNAs and proteins to regulate HCC proliferation and migration, resulting in a poor prognosis in HCC patients. The biological roles of miR-497-5p in HCC development were explored, and we found that miR-497-5p was increased in both HCC tissues and cells, whereas lncRNA XIST was significantly decreased. We also observed that silencing of miR-497-5p could inhibit HCC progression in vitro. In addition, using bioinformatics methods, programmed cell death 4 (PDCD4) was predicted to be the target of miR-497-5p. Thus, we propose that lncRNA XIST inhibits HCC progression by targeting miR-497-5p and PDCD4 in vitro.

## Materials and methods

### Patient specimens

In total, 77 patients with pathological diagnosis of HCC and who underwent hepatectomy at the 1st Affiliated Hospital of Sun Yat-sen University (SYSU) between January 2004 and December 2008 were included in this study. All samples were immediately frozen in a liquid nitrogen tank. The inclusion criteria were as follows: (1) radical resection; (2) no chemotherapy before surgery; (3) no distant metastasis; (4) survival for over 1 month after hepatectomy surgery; and (5) complete clinicopathological and follow-up data are available. In our study, the tumor-node-metastasis (TNM) staging was evaluated based on the American Cancer Joint Commission (AJCC) Cancer Staging Manual, 7th Edition. The basic clinical information of the 77 HCC patients is shown in Table 1. All procedures carried out in studies involving human participants met the ethical standards of the Ethics Committee of the 1st Affiliated Hospital of Sun Yat-sen University and the 1964 Declaration of Helsinki and its subsequent revisions and amendments.

**Table 1:** Correlation between miR-497-5p expression and clinicopathological features of patients with HCC

Clinicopathological variables	n	miR-497-5p expression		p-value
		Low (53)	High (24)	
Gender				
Male	66	47	19	
Female	11	6	5	0.269
Age (years)				
< 50	33	20	13	
≥ 55	44	33	11	0.177
AFP (ng/mL)				
Low, < 200	39	24	15	
High, ≥ 200	38	29	9	0.162
Cirrhosis				
Yes	34	20	14	
No	43	33	10	0.092
HBsAg				
Negative	11	8	3	
Positive	66	45	21	0.761
Tumor size (cm)				
< 5	28	16	14	
≥ 5	49	37	10	0.019
Tumor number				
Solitary	54	34	20	
Multiple (≥ 2)	23	19	4	0.111
PVTT				
No	63	43	20	
Yes	14	10	4	0.545
TNM stage				
I/II	47	27	20	
III/IV	30	26	4	0.011
Edmondson grade				
I/II	30	23	7	
> II	47	30	17	0.026

*AFP* alpha-fetoprotein determination, *PVTT* portal vein tumor thrombus, *HBsAg* hepatitis B surface antigen, *TNM* tumor node metastasis

### Cell culture

Human hepatic carcinoma cell lines (HepG2, HepB3, Huh7, SMMC-7721, MHCC-97L and Bel-7402), an immortalized hepatocyte cell line (LO2) and HEK293T cells were used in this study. All cells were purchased from the Institute of Cell Biology, Chinese Academy of Sciences (Shanghai, China). RPMI 1640 supplemented with 10% fetal bovine serum (FBS) (HyClone, Shanghai, China), 100 U/mL penicillin and 100 μg/mL streptomycin (Gibco) or Dulbecco’s modified Eagle’s medium (DMEM, Sigma) was used as the cell culture medium, and all cells were cultured in a humidified chamber containing 5% CO_2_ at 37 °C.

### Lentiviral vector transfection

The human XIST full complementary DNA (cDNA) was amplified from HCC cells. The shRNA-luciferase (shluc) sequence was designed as a negative control. The target product was subcloned into pcDNA 3.1 (Invitrogen, Carlsbad, CA) using a lentivirus packaging vector and pMD2.G. In the medium containing 800 µg/mL G418 (Sigma-Aldrich), cells stably expressing XIST were cultured. Lipofectamine 2000 (Invitrogen) was used to introduce miR-497-5p mimics, inhibitors or negative controls into cells.

### CCK8 assay

The cells were inoculated in 96-well plates overnight and infected for 48 h with a miRNA-497-5p inhibitor, an empty lentivirus vector, lentivirus (LV)-XIST or a LV negative control (NC) using Dojindo Molecular Technologies (Tokyo, Japan) on days 0, 1, 2, 3 and 4 with a 100-µL cell counting Kit-8 (CCK8). After incubating the cells with the CCK8 reagent for 4 h, the absorbance was measured at 450 nm by enzyme labeling (Bio-Tek, Winooski, VT).

### Transwell invasion assay

A 200-µL cell suspension was loaded into the upper chamber of 24 transwell permeability support chambers with 8-micron pores coated with 1 mg/mL Matrigel (Corning Incorporated, NY). The basement is equipped with 600 µL of RPMI-1640 containing 10% FBS. After that, the cells on the filter surface were fixed with 4% formaldehyde for 15 min, stained with 0.5% crystal violet for 30 min, and then observed using a microscope.

### Scratch wound assay

The cells were inoculated in a 6-well plate, scraped through each hole with the tip of a sterile 10-µL pipette and washed with phosphate buffered saline to remove any debris. After 24 h, the cells that migrated to the empty space were observed.

### qRT-PCR

RNAiso Plus (TaKaRa Biotechnology, Dalian, China) was used to extract total RNA. Prime Script™ RT Master Mix was used to perform RNA reverse transcription. SYBR Premix Ex Taq II (TaKaRa Biotechnology) was used for qPCR. The primers used were as follows: for XIST (sense, 5ʹ-AGCTCCTCGGACAGCTGTAA-3ʹ; antisense, 5ʹ-CTCCAGATAGCTGGCAACC-3ʹ); for PDCD4 (sense, 5ʹ-TCG TCGTTACGATTGGTTAGTC-3ʹ; antisense, 5ʹ-GAAAAATCTCTA ACCCTTCTCGC-3ʹ); for miR-497-5p: (sense, 5ʹ-CCTTCAGCAGCACACTGTGG-3ʹ; antisense, 5ʹ-CAGTGCAGGGTCCGAGGTAT -3ʹ); for U6: (sense, 5′-CTCGCTTCGGCAGCACA-3′; antisense, 5′-TGGTGTCGTGGAGTCG-3′). An Applied Biosystems 7500 Real-Time PCR system (Applied Biosystems, Foster City, CA) was also utilized. The 2^−∆∆Ct^ method was employed to analyze the relative gene expression levels.

### Western blot analysis

Total protein was isolated from the cell lines and then resolved by 10% SDS-PAGE. Isolated proteins were transferred using a polyvinylidene fluoride (PVDF) membrane (Millipore, Billerica, MA). The membrane was incubated with a primary antibody, followed by incubation with secondary antibodies. The main antibodies included anti-PDCD4 (1:2000; Abcam of Cambridge University, Britain) and anti-glyceraldehyde 3-phosphate dehydrogenase (GAPDH) (1:1000, Abcam).

### Flow cytometry

Cells were digested, washed with cold PBS, fixed with 70% cold ethanol and stored at − 20 °C for at least 48 h. Before flow cytometry determination, fixed cells were washed and resuspended in 1 mL of PBS containing 10 mg/mL RNase A and were then incubated for 1 h at 37 °C. Cell suspensions were stained with propidium iodide solution (100 µg/mL) in the dark for 30 min. For each sample, 10,000 events were acquired, and cell cycle determinations were made by a FACS flow cytometer.

### Luciferase reporter gene assay

For the luciferase reporter gene assay, 5 × 10^5^ HEK293T cells were inoculated in a 24-well plate overnight. pmirGLO-PDCD4-WT or pmirGLO-lncRNA XIST-WT reporter plasmids (150 ng each) and their mutant vectors were cotransfected into cells with 50 nM mimic of miRNA-497-5p using Lipofectamine 2000 reagent. After 36 h of cell culture, the firefly and Renilla luciferase activities were determined by a double Luciferase Reporter Analysis System (Promega) based on the manufacturer’s instruction manual. The relative luciferase activity was calculated based on the firefly/Renilla fluorescence ratio.

### Immunohistochemistry

The tissue was fixed by 4% formalin and embedded in paraffin. The endogenous peroxidase activity was blocked, and each slide was subjected to antigen retrieval after peeling and rehydration. The slides were incubated overnight with antibodies against Ki67 (1:500, #ab15580, Abcam) and PDCD4 (1:500, #ab80590, Abcam) at 4 °C. Slides were then incubated with a second antibody coupled with horseradish peroxidase (HRP) at 37 °C for 1 h. The positive immune response rate was determined according to the ratio of positive cells.

### Detection of the xenotransplantation of tumors

The animal experiment procedure was approved by the Animal Ethics Committee of the First Affiliated Hospital of Sun Yat-sen University. One-month-old female BALB/c thymus-free nude mice were purchased from Shanghai Pharmaceutical Research Institute (Shanghai, China). HepG2 cells transfected with LV-XIST or LV-NC were subcutaneously implanted into the lateral abdomen of each nude mouse. After 1 week, the volume of the tumor was estimated using a caliper once a week for 5 weeks. The volume was calculated using the following formula: (mm^3^) = 0.5 × length × width^2^. All mice were euthanized, and the weights of the tumors were measured after 5 weeks. The levels of PDCD4 in the resected tumors were analyzed by Western blot and immunohistochemistry analyses. For immunohistochemical staining, two slices were stained with terminal TUNEL (Yeasen, Shanghai, China) according to the manufacturer’s protocol. The cell nuclei were counterstained with 4,6-diamidino-2-phenylindole (DAPI, Sigma). TUNEL-stained slides were visualized under a fluorescence microscope (Zeiss Axio Observer. Z1).

### Statistical analysis

Student’s t-test was used to analyze the differences between the two experimental groups. One-way ANOVA was used to analyze the differences among more than two different groups. Pearson’s correlation coefficient was used to evaluate the correlations between different groups. χ^2^ test or Fisher’s exact test were used to determine whether the target was correlated with the clinical pathological parameters. SPSS 24.0 software (Chicago, IL, USA) was used for the statistical analysis. A *p *< 0.05 was considered statistically significant.

## Results

### Correlation between miRNA-497-5p expression and clinicopathological features in HCC patients

To illustrate the role of miR-497-5P in the invasive progression of HCC, the expression of miR-497-5p and the basic information, such as clinical pathological features of patients with HCC, are displayed in Table 1. The median age of the patients was 57.5 years (31–75 years), and the median size of tumors was 6 cm (2–18 cm). The results showed that high expression of miRNA-497-5p was correlated with the Edmondson stage (*p* = 0.026), tumor size (*p* = 0.019) and TNM stage (*p* = 0.011). However, there was no significant correlation between miRNA-497-5p and number of tumors (*p* = 0.111), fetoprotein (AFP) (*p* = 0.162), gender (*p* = 0.269), age (*p* = 0.177), alpha cirrhosis (*p* = 0.092), HBV infection (*p* = 0.761) or portal vein cancer thrombus (PVTT) (*p* = 0.545).

### High levels of miR-497-5p predict a poor prognosis

**Fig. 1:**
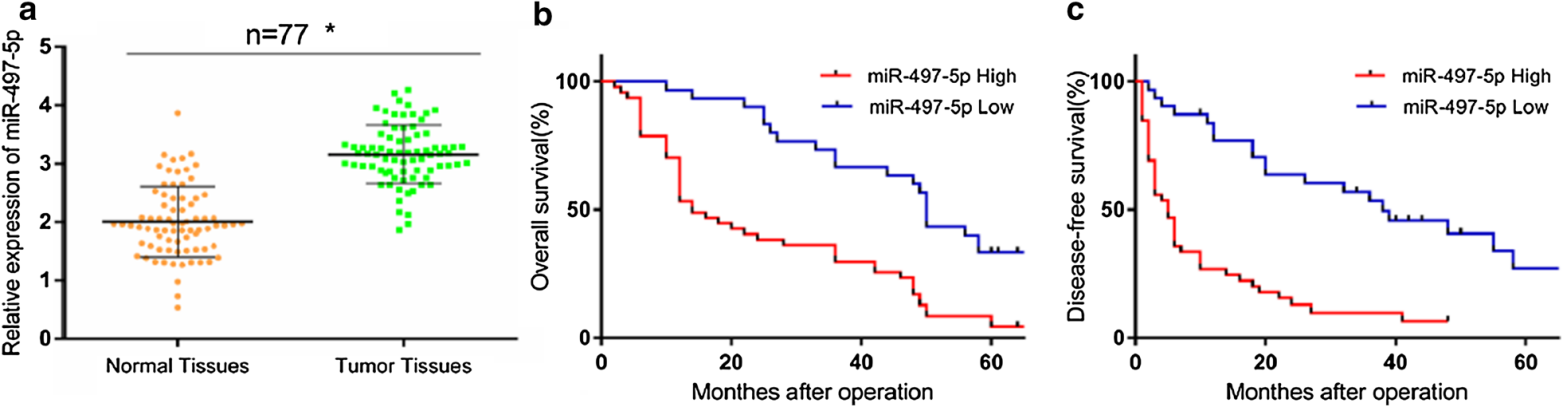
miR-497-5p was increased in HCC and correlated with prognosis in HCC patients. **a** Expression levels of miR-497-5p in HCC tissues and adjacent normal tissues were measured by qRT-PCR. **b** The overall survival of HCC patients with high or low expression of miR-497-5p were evaluated by Kaplan–Meier analysis. **c** The disease-free survival of HCC patients with high or low expression of miR-497-5p were evaluated by Kaplan–Meier analysis. Error bars represent the mean ± SD from three independent experiments. *p < 0.05. *miR* microRNA-497-5p

We further studied the relationship between the level of miRNA-497-5p and survival time in 77 HCC patients to clarify the prognostic value of miRNA-497-5p. The level of miRNA-497-5p was detected by qRT-PCR, and the results showed that compared with the adjacent normal tissues, miRNA-497-5p was significantly upregulated in the HCC tissues (Fig. 1a). Significant differences were found in the overall survival (OS) and disease-free survival (DFS) between different miRNA-497-5p expression groups (the high and low group) (*p* < 0.001 and *p *< 0.001), as shown in Fig. 1b, c. In addition, univariate and multivariate Cox proportional risk regression analyses were used to determine the independent prognostic factors of OS and DFS in HCC patients. The results showed that the Edmondson grade (hazard ratio [HR]: 2.768; 95% confidence interval [CI] 1.206–6.352 for III + IV vs. I + II, *p *= 0.016) and lower miRNA-497-5p expression (HR 0.016; 95% CI 0.242–0.767, *p *= 0.015) were significant independent prognostic factors of OS (Table 2). In addition, the results demonstrated that the Edmondson grade (HR, 5.442; 95% CI 2.778–9.714, p = 0.001) and lower miRNA-497-5p expression (HR, 0.090; 95% CI 0.018–0.461, *p *= 0.004) were significant prognostic factors of DFS in patients with HCC (Table 3).

**Table 2:** Univariate and multivariate Cox regression analyses of risk factors associated with overall survival

Variables	Univariate analysis			Multivariate analysis		
	HR	95% CI	p-value	HR	95% CI	p-value
miR-497-5p expression (low vs. high)	4.603	1.914–9.465	0.001	4.116	1.542–8.767	0.005
Gender (male vs. female)	0.656	0.325–1.326	0.241			
Age (< 50 vs. ≥ 55)	0.609	0.370–1.005	0.052			
HBsAg (negative vs. positive)	0.918	0.481–1.753	0.796			
Cirrhosis (yes vs. no)	1.435	0.886–2.325	0.142			
AFP (< 200 ng/mL vs. ≥ 200 ng/mL)	1.766	1.092–2.857	0.020	1.548	0.924–2.592	0.097
Tumor size (< 5 cm vs. ≥ 5 cm)	1.651	1.007–2.707	0.047	0.718	0.387–1.331	0.293
Tumor number (single vs. multiple)	2.184	1.296–3.680	0.003	1.105	0.585–2.088	0.758
PVTT (no vs. yes)	3.487	1.852–6.564	0.001	1.229	0.537–2.816	0.626
TNM stage (I/II vs. III/IV)	3.331	1.989–5.578	0.001	2.401	0.791–2.285	0.171
Edmondson grade (I/II vs. > II)	4.384	2.561–7.505	0.001	2.768	1.206–6.352	0.016

*AFP* alpha-fetoprotein determination, *PVTT* portal vein tumor thrombus, *HBsAg* hepatitis B virus, *TNM* tumor node metastasis, *HR* hazard ratio, *CI* confidence interval

**Table 3:** Univariate and multivariate Cox regression analyses of risk factors associated with disease-free survival

Variables	Univariate analysis			Multivariate analysis		
	HR	95% CI	p-value	HR	95% CI	p-value
miR-497-5p expression (high vs. low)	3.358	1.516–7.408	0.001	3.197	1.264–6.121	0.004
Gender (male vs. female)	0.379	0.116–1.237	0.108			
Age (< 50 vs. ≥ 55)	0.501	0.246–1.024	0.058			
HBsAg (negative vs. positive)	1.104	0.429–2.838	0.838			
Cirrhosis (no vs. yes)	2.181	1.092–4.355	0.027	1.065	0.455–2.497	0.884
AFP (< 200 ng/mL vs. ≥ 200 ng/mL)	2.311	1.182–4.518	0.014	0.951	0.371–2.135	0.616
Tumor size (< 5 cm vs. ≥ 5 cm)	3.523	1.595–7.782	0.002	1.584	0.591–4.245	0.360
Tumor number (single vs. multiple)	1.652	0.818–3.337	0.162			
PVTT (no vs. yes)	3.971	1.798–8.768	0.001	1.651	0.759–3.590	0.206
TNM stage (I/II vs. III/IV)	2.945	1.511–5.739	0.002	1.570	0.609–4.046	0.351
Edmondson grade (I/II vs. > II)	3.384	1.419–9.660	< 0.01	5.442	2.778–9.714	0.001

*AFP* alpha-fetoprotein determination, *PVTT* portal vein tumor thrombus, *HBsAg* hepatitis B surface antigen, *TNM* tumor node metastasis, *HR* hazard ratio, *CI* confidence interval

### miR-497-5p was upregulated in HCC and could promote cell proliferation and migration in HCC

**Fig. 2:**
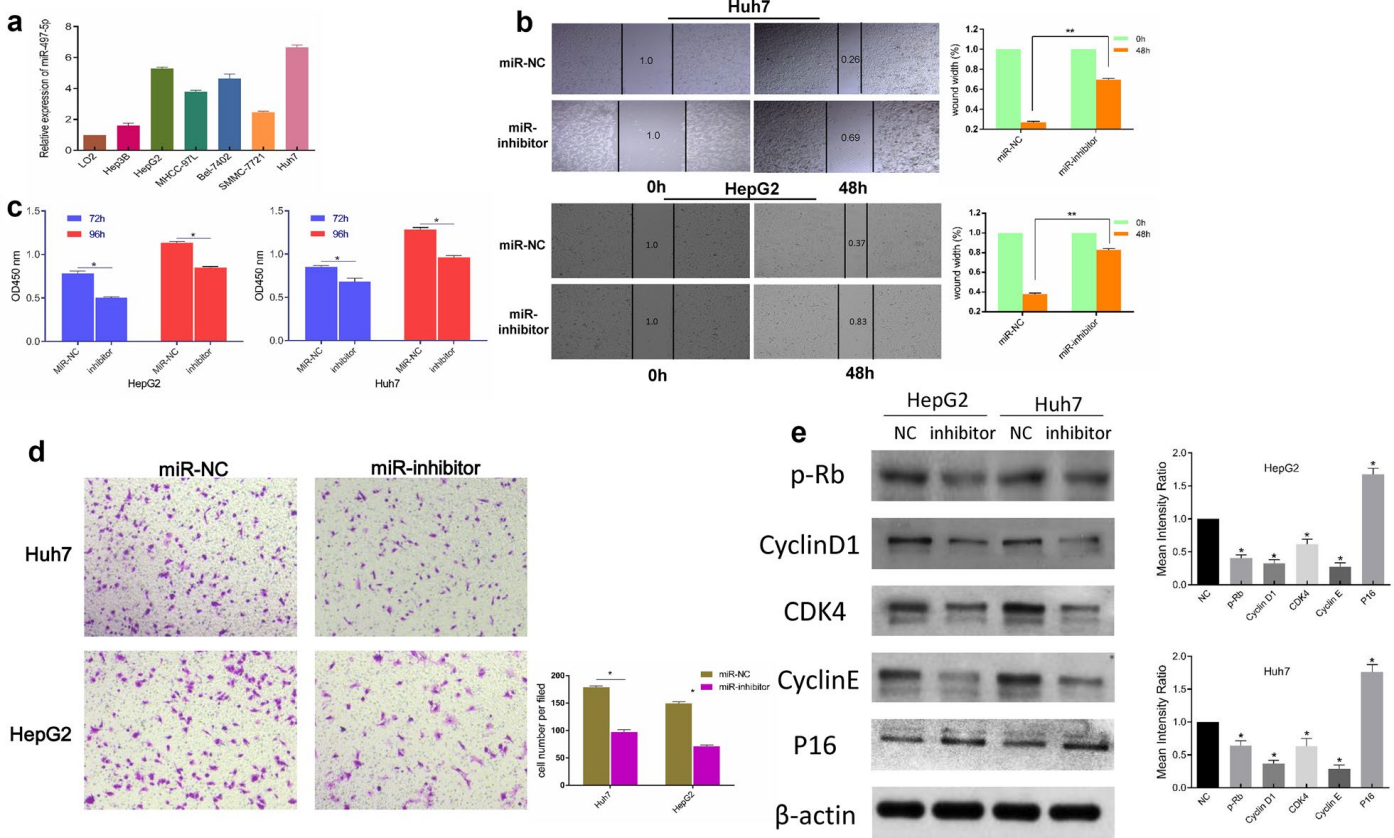
miR-497-5p was upregulated in HCC cell lines and promoted the proliferation and migration of HCC cells. **a** The level of miR-497-5p in HCC and L02 cell lines. **b** Wound healing assay was performed to measure the effect of miR-NC (cells transfected with empty lentiviral vectors served as a negative control) and miR-inhibitor (cells transfected with lentiviral vectors with short hairpin RNA targeting miR-497-5p) on the migration of Huh7 and HepG2 cells. **c** CCK-8 assay after 72 h and 96 h of treatment with miR-inhibitor in Huh7 and HepG2 cells. **d** The invasion ability of Huh7 and HepG2 cells was measured by transwell assay (original magnification, ×200). **e** Western blot analysis showed that miR-497-5p promoted the expression of p-Rb, Cyclin D1, CDK4 and Cyclin E and inhibited the expression of P16. Error bars represent the mean ± SD from three independent experiments. *p < 0.05, **p < 0.01. *miR* microRNA-497-5p

Given the increased expression of miR-497-5p in the HCC tissues, we explored the expression levels of miR-497-5p in HCC cell lines (Huh7, Bel-7402, HepG2, Hep3B, MHCC-97L and SMMC-7721) and the hepatocyte cell line L02 (HL-7702) and discovered that miR-497-5p was upregulated in the HCC cell lines (shown in Fig. 2a). Then, we transfected Huh7 and HepG2 cells with a miR-497-5p inhibitor to assess the biological effect of miR-497-5p in HCC cells. Wound healing and CCK8 assays revealed that the miR-497-5p-inhibitor suppressed the proliferative capacity of the HepG2 and Huh7 cells (Fig. 2b, c). In addition, compared with the control group, the migration of the Huh7 and HepG2 cells transfected with miRNA-497-5p-inhibitors was also decreased (Fig. 2d). To explore the roles in cellular activity, the expression levels of several cell cycle regulatory markers were further examined by Western blot analysis. The results showed that Cyclin D1, CDK4 and Cyclin E expression levels were inhibited while that of P16 was elevated in the miR-497-5p inhibitor group. In comparison, miR-NC significantly increased the expression levels of Cyclin D1, CDK4 and Cyclin E and decreased the expression of P16 (Fig. 2e).

### PDCD4 is a direct target of miR-497-5p

**Fig. 3:**
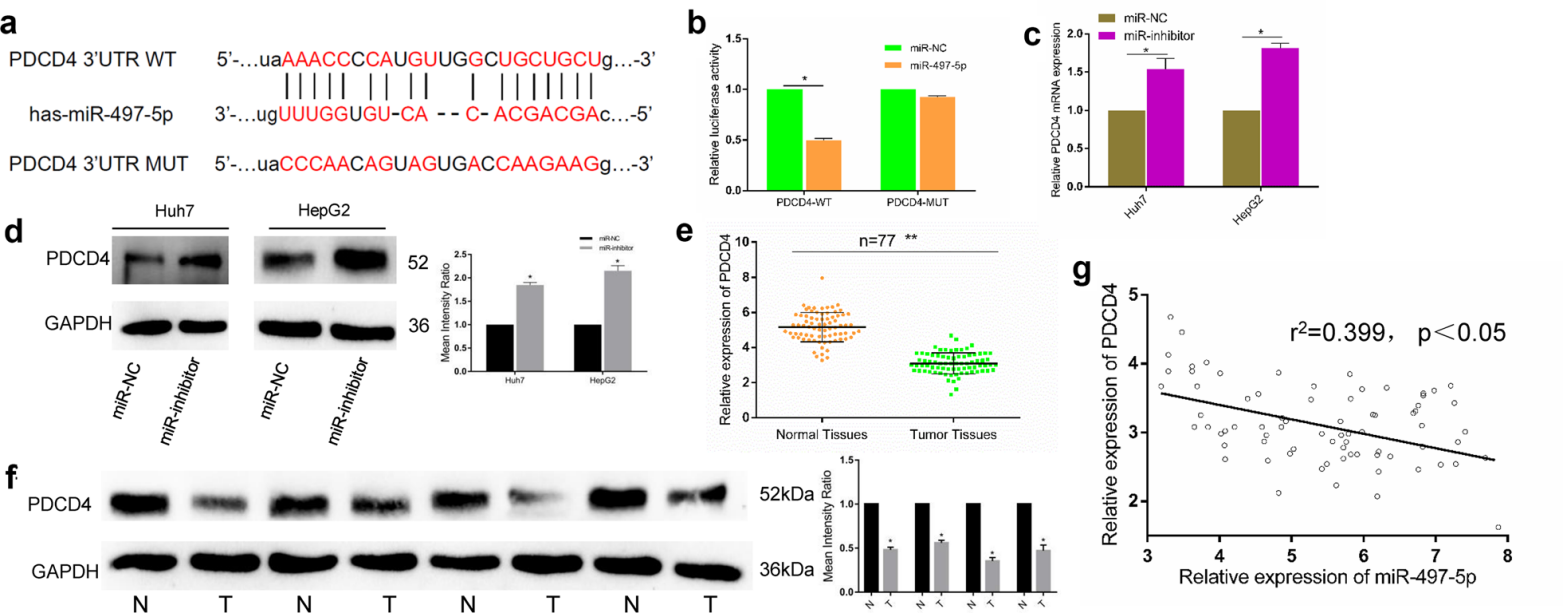
PDCD4 was a direct target of miR-497-5p and was downregulated in HCC. **a** The putative binding sequence for miR-497-5p in the 3′-UTR of PDCD4. **b** Luciferase activity assay revealed that the miR-497-5p mimic suppressed PDCD4 3′-UTR WT luciferase activity, while it had no effect on PDCD4 Mut luciferase activity in HCC cells. **c** The mRNA expression of PDCD4 was examined in Huh7 and HepG2 cells after transfection with miR-497-5p inhibitor. **d** The protein expression of PDCD4 was examined by western blot in Huh7 and HepG2 cells after transfection with miR-497-5p inhibitor. **e** qRT-PCR was used to measure the expression of PDCD4 in HCC tumor tissues and matched normal tissues. **f** Low expression of PDCD4 was observed in HCC tissues by western blot. **g** The expression of PDCD4 was negatively correlated with the expression of miR-497-5p in HCC. Error bars represent the mean ± SD from three independent experiments. *p < 0.05, **p < 0.01. *IHC* immunohistochemistry, *NC* negative control, *Mut* mutated, *UTR* untranslated region, *WT* wild type

To identify the underlying mechanism of miR-497-5p in HCC, the miRanda database was searched to make a prediction of the potential target for miR-497-5p. PDCD4 was suggested as a potential target of miRNA-497-5p due to the presumptive binding sequence of microRNA-497-5p in its 3′-UTR (shown in Fig. 3a). To confirm this prediction, double luciferase assay assays were performed. The results are shown in Fig. 3b; miR-497-5p inhibited the relative luciferase activity in cells containing the wild-type (WT) 3′-UTR of the PDCD4 construct but did not alter that in cells containing the mutated (Mut) 3′-UTR of the PDCD4 construct. To further verify that PDCD4 is a target of miRNA-497-5p, we surveyed the expression of PDCD4 in miR-497-5p- or NC miRNA-transfected cells. As expected, the protein expression of PDCD4 was increased by the miR-497-5p-inhibitor (Fig. 3c, d), revealing that PDCD4 is a direct target of miR-497-5p in HCC.

### PDCD4 downregulates and inhibits the proliferation and migration of HCC cells

**Fig. 4:**
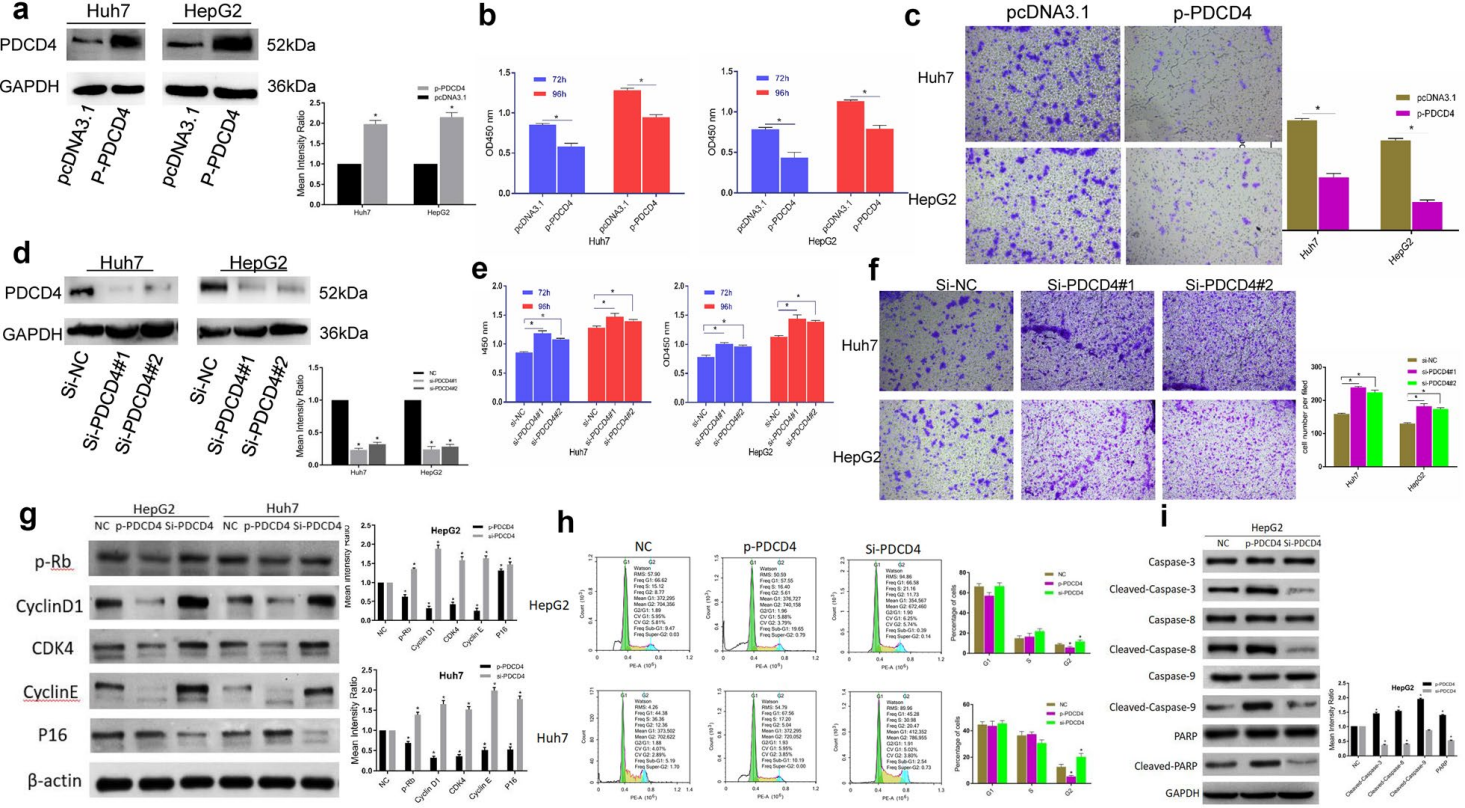
PDCD4 inhibited the proliferation and migration of HCC cells. **a** Overexpression efficacy of PDCD4 in Huh7 and HepG2 cells by western blot. **b**, **c** PDCD4 overexpression inhibited the proliferation and migration of Huh7 and HepG2 cells by CCK8 and transwell assays. **d** Knockdown efficacy of PDCD4 in Huh7 and HepG2 cells by western blot analysis. **e**, **f** Knockdown of PDCD4 enhanced the proliferation and migration of Huh7 and HepG2 cells by CCK8 and transwell assays. **g** Western blot analysis of cell cycle regulatory proteins in different HCC cells after transfection with PDCD4 and siPDCD4. **h** The cell cycle distribution of HepG2 and Huh7 cells after PDCD4 upregulation or downregulation was analyzed by flow cytometry. **i** Gray value of cleaved caspase 3, 8, 9 and PARP protein bands in HepG2 cells after transfection with PDCD4 and siPDCD4 examined by Western blot analysis. Error bars represent the mean ± SD from three independent experiments. *p < 0.05

Since PDCD4 was found to be a direct target of miR-497-5p, the expression levels of PDCD4 in 77 pairs of HCC samples were measured by qRT-PCR. Compared with the adjacent normal tissues, PDCD4 was downregulated in 57 (74.0%) HCC tissues (p < 0.001, Fig. 3e). Western blot analysis further confirmed the significant downregulation of PDCD4 in HCC (Fig. 3f). Additionally, we examined the expression levels of PDCD4 and miR-497-5p and found that they were negatively correlated (Fig. 3g). Subsequently, we overexpressed PDCD4 in HepG2 and Huh7 cells (Fig. 4a) and discovered that overexpression of PDCD4 suppressed cell proliferation and migration (Fig. 4b, c). Furthermore, knocking down PDCD4 enhanced the cell proliferation and migration of the HepG2 and Huh7 cells (Fig. 4d, f). To investigate the effects of PDCD4 on apoptosis and the cell cycle in HCC cells, we detected the expression levels of Cyclin D1, CDK4, Cyclin E, P16 and apoptosis markers such as cleaved caspase 3, caspase 8, caspase 9, poly ADP ribose polymerase (PARP) in HepG2 and Huh7 cells with upregulation or downregulation of PDCD4. Flow cytometry was then utilized for cell cycle analysis. The results of this experiment showed that si-PDCD4 significantly increased the expression levels of Cyclin D1, CDK4 and Cyclin E and decreased the expression of P16 (Fig. 4g). As shown in Fig. 4h, cells were blocked in G2 phase after interfering with PDCD4. The si-PDCD4 group also exhibited a decreased apoptotic rate and reduced protein expression levels of cleaved caspase 3, 8 and 9 and PARP (Fig. 4i). All of these results indicate that miR-497-5p can promote cell proliferation and migration in HCC by targeting PDCD4.

### XIST and miR-497-5p are negatively related in HCC

**Fig. 5:**
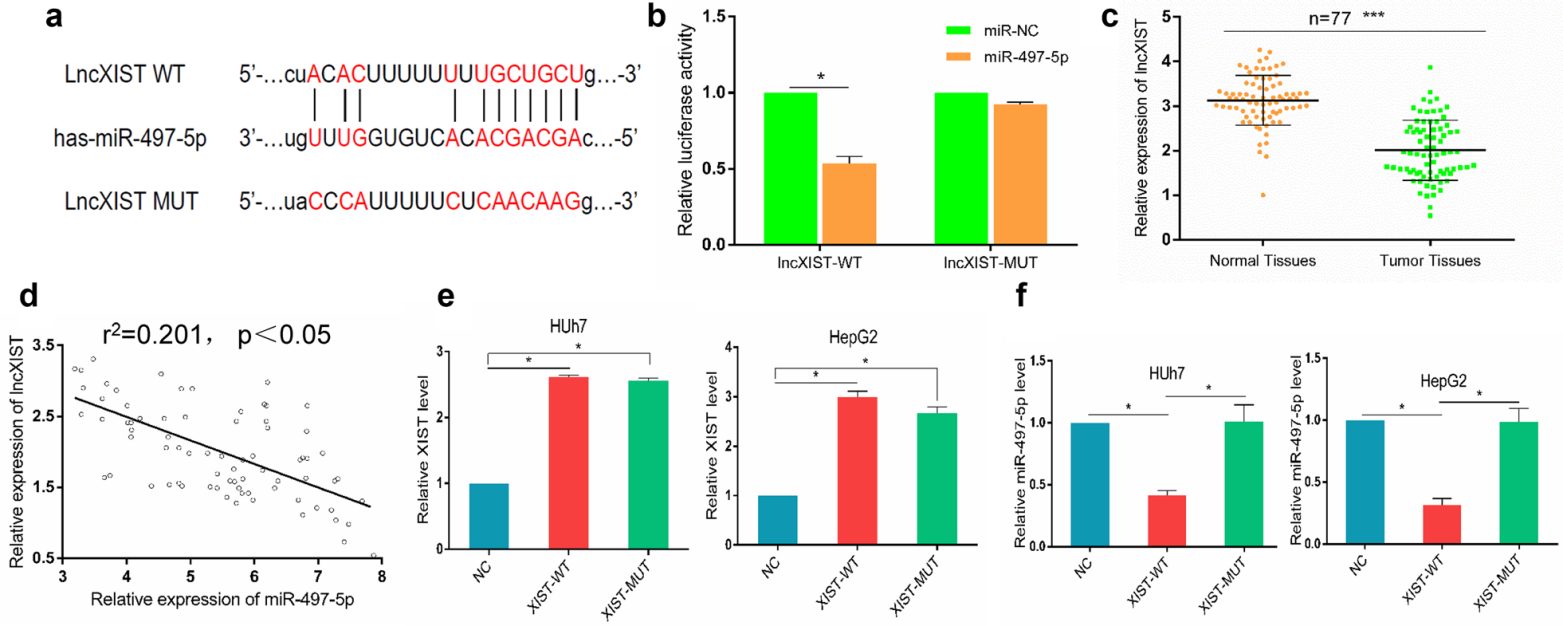
XIST negatively regulated miR-497-5p in HCC. **a** Wild-type (WT) or mutated (MUT) XIST fragments containing the predicted miR-497-5p binding sites. **b** Luciferase activity assay revealed that the miR-497-5p mimic suppressed XIST-WT luciferase activity, while it had no effect on XIST-MUT luciferase activity in HCC cells. **c** The expression of XIST in 77 paired HCC tissues and their matched adjacent tissues was examined by qRT-PCR. **d** The correlation between XIST and miR-497-5p in HCC tissues. **e** The expression levels of XIST in Huh7 and HepG2 cells transfected with XIST-WT, XIST-MUT and negative control were examined using qRT-PCR. **f** The expression levels of miR-497-5p in Huh7 and HepG2 cells transfected with XIST-WT, XIST-MUT and negative control were determined by qRT-PCR. Error bars represent the mean ± SD from three independent experiments. *p < 0.05, ***p < 0.001. *NC* negative control, *MUT* mutated, *WT* wild type

To investigate the underlying biological mechanism by which XIST has an effect on HCC development, we carried out a bioinformatics analysis. We used miRanda (http://www.microrna.org) and StarBase v3.0 (http://starbase.sysu.edu.cn/) to predict possible lncRNAs interacting with miR-497-5p. The lncRNA XIST, which contained complementary sequences to the seed region of miR-497-5p, was predicted. The predicted binding sites of XIST and miR-497-5p are shown in Fig. 5a. To validate the interaction between miR-497-5p and XIST, we carried out luciferase reporter assays. The plasmids of pmirGLO-lncRNA XIST-WT and pmirGLO-lncRNA XIST-MUT were cotransfected into HepG2 and Huh7 cells with miRNA-497-5p or miRNA-control. The results indicated that ectopic expression of microRNA-497-5p resulted in a significant downregulation in the luciferase activity of XIST-WT, but not XIST-MUT, in Huh7 and HepG2 cells (Fig. 5b). The qRT-PCR results showed that lncRNA XIST was significantly downregulated in the 77 HCC tissues compared with the matched adjacent normal tissues (Fig. 5c). In addition, a significant negative correlation between the expression of miR-497-5p and XIST was found in HCC tissues (Fig. 5d). To further pinpoint the regulatory relationship between miR-497-5p and XIST, we transfected HepG2 and Huh7 cells with XIST-mimic, XIST-MUT, XIST-WT or respective controls. XIST was obviously increased after transfection with the XIST-mimic (Fig. 5e). Moreover, the level of miR-497-5p was considerably elevated in HepG2 and Huh7 cells following the introduction of XIST-WT, but there was no distinct change in the cells treated with XIST-MUT (Fig. 5f). These data indicate that a direct interaction exists between XIST and miR-497-5p in HCC.

### LncRNA XIST regulates the proliferation and migration of HCC cell lines through the miRNA-497-5p axis

**Fig. 6:**
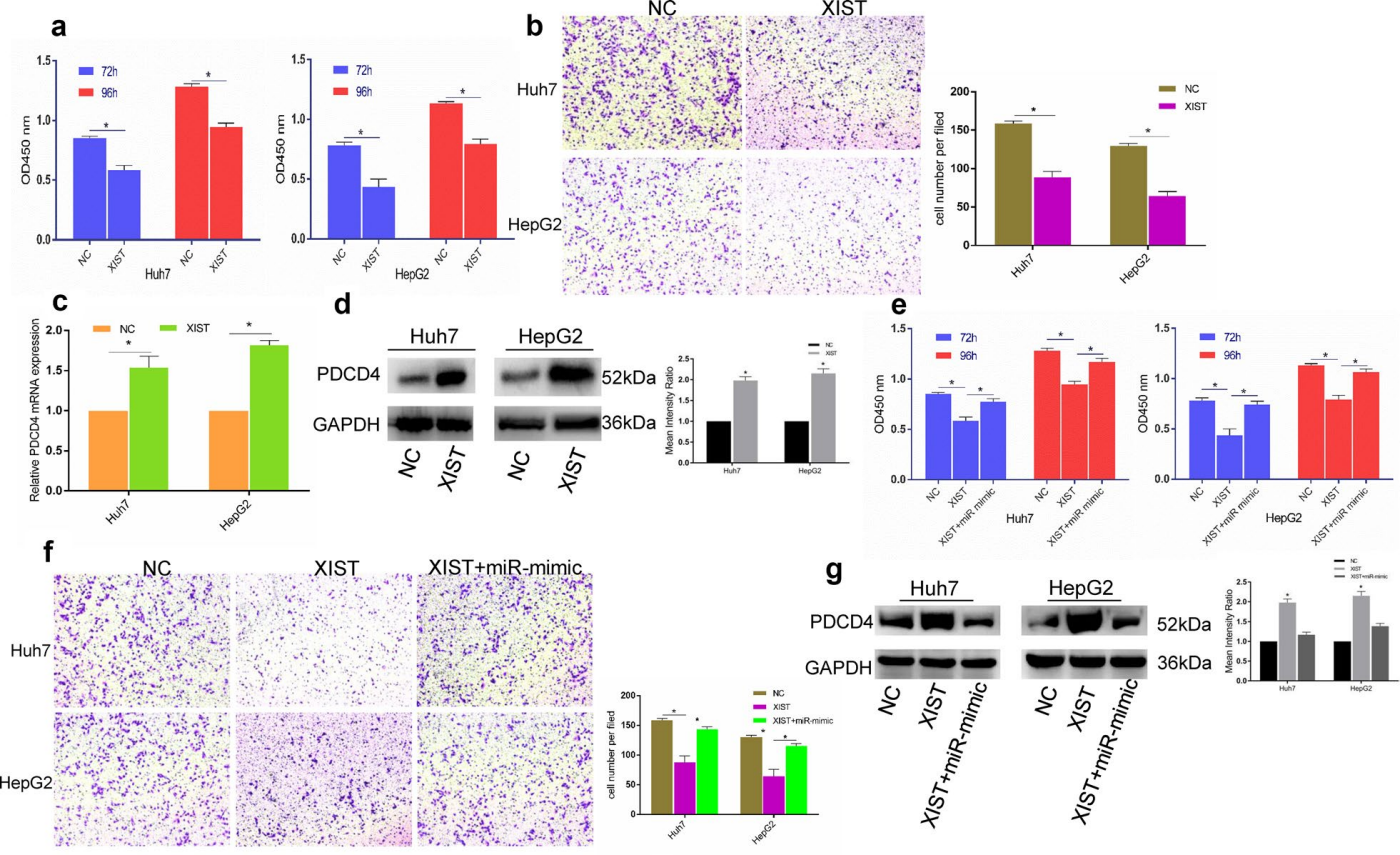
lncRNAXIST regulated the proliferation and migration of HCC cells via the miR-497-5p-PDCD4 axis. **a** The effects of lncRAXIST overexpression on Huh7 and HepG2 cells by CCK-8 assay. **b** lncRNAXIST overexpression inhibited the migration of Huh7 and HepG2 cells. **c**, **d** lncRNAXIST overexpression increased PDCD4 mRNA and protein expression in Huh7 and HepG2 cells. **e**, **f** The miR-497-5p mimic rescued XIST overexpression -induced suppression of proliferation and migration in both Huh7 and HepG2 cells. **g** Overexpression of PDCD4 induced by XIST-mimic could be rescued by miR-497-5p overexpression. Error bars represent the mean ± SD from three independent experiments. *p < 0.05. *IHC* immunohistochemistry, *NC* negative control

After confirming that lncRNA XIST could bind miR-497-5p and is positively correlated with PDCD4 expression, we hypothesized that lncRNA XIST plays an equally vital role in HCC. We then overexpressed lncRNA XIST and found that the proliferation and migration of the HepG2 and Huh7 cells were obviously suppressed by LV-XIST, but there was no difference in the cells transfected with the XIST negative control (NC) (Fig. 6a, b).

If a lncRNA serves as a molecular sponge of miRNA, then its upregulation could lead to an elevation in the miRNA targets. To determine whether lncRNA XIST regulated the proliferation and migration in HepG2 and Huh7 cells through targeting PDCD4 by sponging miR-497-5p, we overexpressed lncRNA XIST and discovered that both the PDCD4 gene and protein expression levels were increased (Fig. 6c, d). We conducted rescue experiments to determine whether lncRNA XIST regulates cell proliferation and PDCD4 expression via miR-497-5p in HCC. The reductions in the proliferation and migration of the Huh7 and HepG2 cells generated by lncRNA XIST overexpression could be rescued by the miR-497-5p mimic (Fig. 6e, f). The inhibition of PDCD4 by lncRNA XIST could also be rescued by overexpressing miR-497-5p (Fig. 6g). Altogether, the above data indicate that lncRNA XIST regulates the cell proliferation and migration in HCC through the miR-497-5p-PDCD4 axis.

### Overexpression of lncRNA XIST inhibited tumor growth in vivo

**Fig. 7:**
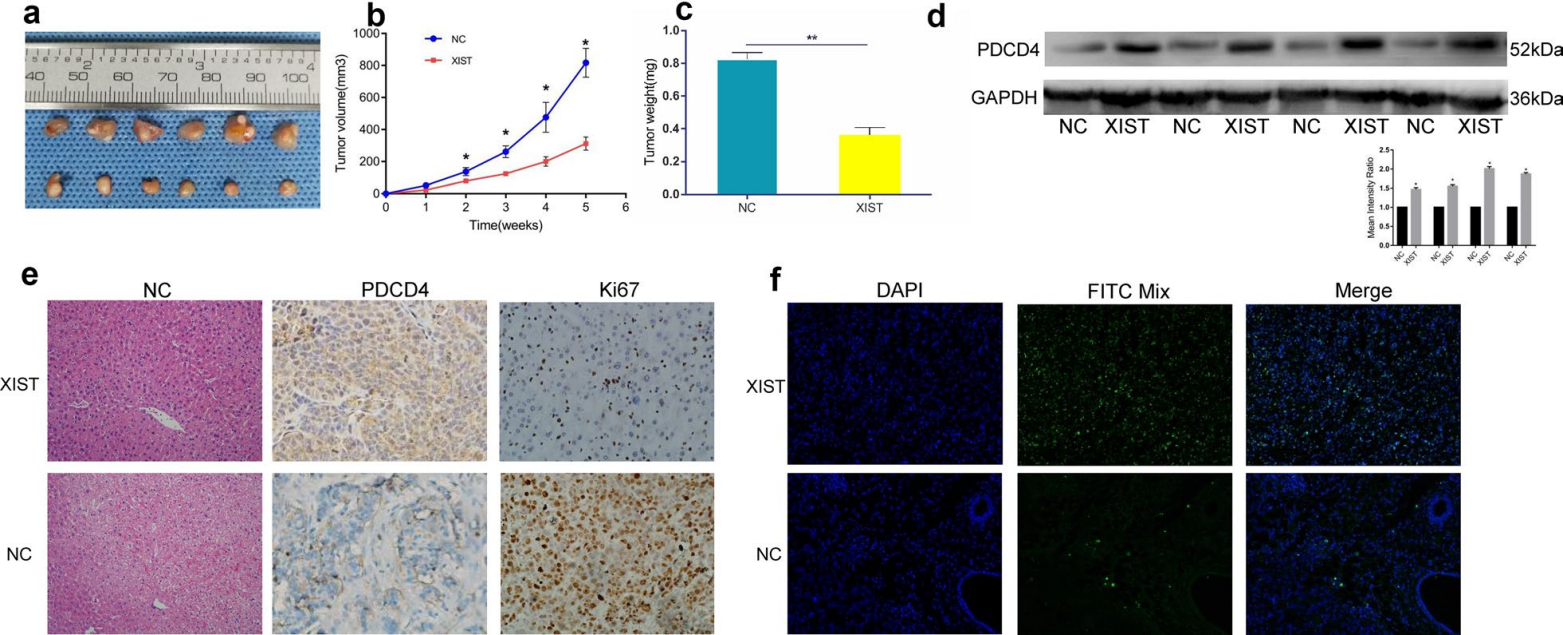
Overexpression of lncRNAXIST inhibited tumor growth in vivo. **a** The xenograft tumors were obviously inhibited by lncRNAXIST. **b**, **c** Tumor volume and tumor weight of the xenografts were significantly suppressed by lncRNAXIST-mimic. **d** lncRNAXIST-mimic upregulated PDCD4 expression in vivo by western blot analysis. **e** The expression of PDCD4 and Ki67 in the xenograft tumors was examined by IHC. Error bars represent the mean ± SD from three independent experiments. **f** Terminal deoxynucleotidyl transferased UTP nick end labeling (TUNEL) of specific tumor tissues at 21 days post-treatment. Green fluorescence indicated TUNEL-positive apoptotic cells; blue fluorescence indicated DAPI-stained nuclei. **p < 0.01. *IHC* immunohistochemistry, *NC* negative control, *Mut* mutated, *UTR* untranslated region, *WT* wild type

**Fig. 8:**
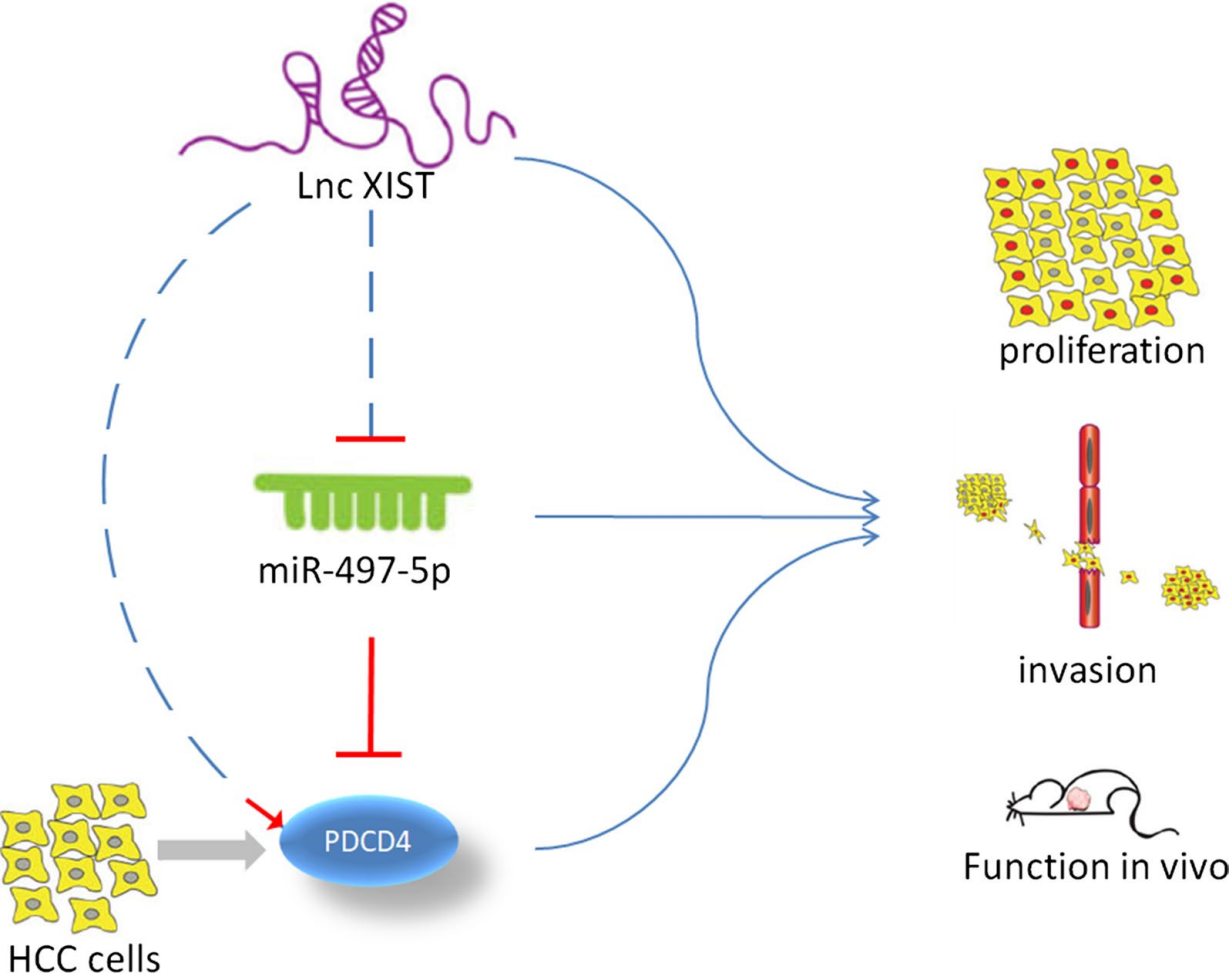
Schemtic diagram shows how XIST regulates HCC cells proliferation and invasion by miR-497-5p as a ceRNA

We verified that lncRNA XIST overexpression inhibited the viability of Huh7 and HepG2 cells in vitro. Therefore, we propose that lncRNA XIST performs the same function in vivo. Twelve mice were randomly divided into two groups: six mice were injected with HepG2 cells with stable lncRNA XIST overexpression (LV-lncRNA XIST-HepG2), and six mice were injected with cells transfected with the control vector (LV-NC-HepG2). The tumor volume was measured weekly for consecutive weeks. After 5 weeks, the mice were sacrificed by anesthesia, and the xenograft tumors were collected for further analysis. Consistent with the in vitro results, the lncRNA XIST overexpression inhibited HepG2 xenograft growth in vivo (Fig. 7a, c). The proliferation marker Ki67 was reduced in the LV-lncRNA XIST-HepG2 xenografts compared with that in the LV-NC-HepG2 xenografts, indicating that lncRNA XIST inhibited tumor growth. Furthermore, Western blot and IHC analyses further confirmed the overexpression of PDCD4 in the LV-lncRNA XIST-HepG2 xenografts in vivo (Fig. 7d, e). Consistently, the terminal deoxynucleotidyl transferase (TdT)-mediated dUTP nick end labeling (TUNEL) staining results were in agreement with the above results: many more FITC-positive apoptotic cells were observed in the XIST-treated group (Fig. 7f). A schematic diagram is depicted in Fig. 8 and summarizes the key findings of our study. Overall, our findings indicate that lncRNA XIST can regulate HCC tumor growth through the miR-497-5p-PDCD4 axis.

## Discussion

The prognosis of HCC patients is typically poor. The main cause of the poor prognosis of these patients is the high possibility of metastasis and recurrence after operation. Previous studies have shown that advanced TNM staging is a vital independent predictor of poor prognosis in HCC. In this study, we also found that a late TNM stage and high expression of miRNA-497-5p were independent prognostic factors of OS and DFS in HCC through a Cox proportional hazard analysis. Additionally, the results of Kaplan–Meier curve analysis showed that patients with high levels of miRNA-497-5p had a worse prognosis than those with low miRNA-497-5p expression. In addition, a higher level of miR-497-5p was associated with malignant behavior in HCC (Table 1), including a larger tumor size, later TNM stage, and higher PVTT and Edmondson grade (Table 2). These findings suggest that the highly expressed miRNA-497-5p is involved in the progression of HCC.

Recent studies have shown that ncRNAs could serve as molecular markers of cancers, including HCC [25, 26]. Therefore, it is important to clarify the roles of miRNAs and lncRNAs in the prognosis of HCC. In this study, miRNA-497-5p was upregulated in HCC cells, while lncRNA XIST was downregulated. The overexpression of XIST could inhibit the development of HCC and rescue the miR-497-5p mimic-induced progression of HCC in vitro. In addition, using bioinformatics methods, we predicted that PDCD4 is a target of miRNA-497-5p. Therefore, we propose that the XIST/miR-497-5p/PDCD4 axis participates in the development of HCC. We found that the level of lncRNA XIST was lower in HCC cells and that overexpression of lncRNA XIST inhibited the progression of HCC. The above results indicate that lncRNA XIST plays a major role in inhibiting HCC development.

Studies have demonstrated that XIST could modulate proliferation and apoptosis in osteoarthritis chondrocytes [27]. In addition, it has been reported that the level of lncRNA XIST in many cancer patients is reduced. For example, in ovarian cancer, the upregulation of lncRNA XIST has anticancer effects due to the inverse downregulation of has-miR-214-3p [28]. In breast cancer, XIST could inhibit proliferation and migration by activating MSN-c-Met and reprogramming microglia to promote brain metastasis [29]. Importantly, the elevation in miR-497-5p may contribute to the XIST-mediated inhibition of liver cancer cell growth. In the current study, our findings provide novel evidence to support this action. This affirmation stems from several findings: (1) XIST is negatively associated with miR-497-5p levels in human HCC tissues and cells; (2) XIST suppressed miR-497-5p expression by targeting PDCD4; and (3) XIST increased the expression of PDCD4 by decreasing miR-497-5p in human liver cancer tissues.

PDCD4 is involved in regulating apoptosis, is located on human chromosome 10q25.2 and is considered a novel tumor suppressor gene [30]. While the PDCD4 protein is usually located in the nucleus, when the cell microenvironment changes, such as during malignant proliferation, PDCD4 can be transferred to the cytoplasm through nuclear export signals [31]. PDCD4 can also bind ribosomes directly, affect the posttranscriptional translation process, and lead to cell apoptosis in cancer [32]. In this study, we predicted that PDCD4 was a target gene of miRNA-497-5p by bioinformatics and that the expression level of PDCD4 could be inhibited by miR-497-5p. In addition, we found that XIST could attenuate the level of PDCD4 and that PDCD4 expression could be inhibited by overexpressed miR-497-5P in HCC cells.

## Conclusions

Altogether, the results of this study indicate that XIST may have an inhibitory effect on HCC. In this work, we confirmed the potential mechanism of the XIST/miR-497-5P/PDCD4 axis in HCC cells and determined that XIST overexpression could inhibit the progression of HCC. In addition, we found a negative correlation between XIST and miRNA-497-5p. We focused on PDCD4 because it is a target of miR-497-5p. Our findings reveal that the XIST/miR-497-5p/PDCD4 axis participates in the development of HCC and that XIST may be considered a potential biomarker of HCC.

## Data Availability

The data used and analyzed in this study are available from the corresponding author upon request.

## Abbreviations

HCC: hepatocellular carcinoma
lncRNAs: long noncoding RNAs
3′-UTR: 3′-untranslated region
HBV: hepatitis B virus
IHC: immunohistochemistry
AFP: alpha-fetoprotein
TNM: tumor-node-metastasis
qRT-PCR: quantitative real-time PCR
